# Assessment of psychosocial risks in a cable manufacturing industry

**DOI:** 10.1192/j.eurpsy.2026.11301

**Published:** 2026-07-31

**Authors:** W. Ayed, M. Mersni, Y. Sellaouti, S. Chemingui, W. Riahi, G. Bahri, M. Bani, H. Nefzi, N. Ladhari

**Affiliations:** 1 Occupational Medicine Department, Charles Nicolle Hospital, Tunis; 2 Psychiatric department, Razi hospital, Mannouba, Tunisia

## Abstract

**Introduction:**

The cable manufacturing industry is characterised by precision requirements, sustained production rates and sometimes demanding working conditions which can exposes workers to various psychosocial risks.

**Objectives:**

To determine the psychosocial factors affecting workers in the cable manufacturing industry.

**Methods:**

Cross-sectional study involving workers in a cable manufacturing industry in Tunisia, conducted over a two-month period from 1 January 2025 to 1 March 2025. The psychosocial factors were analysed using a tool for collecting and analysing risk factors, the ‘MSD questionnaire’, in its new version validated by the INRS (French National Research and Safety Institute) and comprising five parts: general information on the characteristics of the operators, MSD complaints, the main symptoms of stress, psychosocial factors, and work experience.

**Results:**

The average age of our study population was 38±7 years. Women predominated in 98% of cases. The average length of service was 9±5 years. Operators reported that the most demanding shift was the night shift (76%). The psychosocial risk factors of occupational origin reported by workers were: the fast pace of tasks (41%), the required output (29%), and time pressure (53%). Regarding the qualitative requirements of the job, workers complained about tasks requiring attention (23%) and concentration (18%). They reported that they had no autonomy at work in 53% of cases and that they did not participate in the organisation of work in 30% of cases. They reported that they had good social relationships with colleagues (92%) and with their line manager (41%).

**Conclusions:**

In our study, we highlight the prevalence of psychosocial risks among workers in the cable industry, emphasising the need for targeted interventions to improve working conditions, particularly by promoting greater autonomy and increased worker participation in task organisation, in order to reduce the impact of occupational stress factors.

**Disclosure of Interest:**

None Declared

